# Thermal Properties of Warm- versus Heated-Needle Acupuncture

**DOI:** 10.1155/2022/4159172

**Published:** 2022-02-28

**Authors:** Hyo-Rim Jo, Seong-Kyeong Choi, Won-Suk Sung, Seung-Deok Lee, Byung-Wook Lee, Eun-Jung Kim

**Affiliations:** ^1^Department of Acupuncture & Moxibustion, College of Korean Medicine, Dongguk University Graduate School, Seoul, Republic of Korea; ^2^Department of Acupuncture & Moxibustion, Dongguk University Bundang Oriental Hospital, Seongnam, Republic of Korea; ^3^Institute of Oriental Medicine, College of Korean Medicine, Dongguk University, Goyang, Republic of Korea; ^4^Department of Literature and Medical History, College of Korean Medicine, Dongguk University, Gyeongju, Republic of Korea; ^5^Department of Acupuncture & Moxibustion, Dongguk University, Seoul, Republic of Korea

## Abstract

**Background:**

Warm-needle acupuncture (WA) and fire-needle acupuncture are treatment techniques that use the combination of acupuncture and thermal stimulation. In clinical practice, a new method of fire-needle acupuncture called “heated-needle acupuncture (HA)” has been proposed, wherein the needle is directly heated after insertion. WA and HA share similarities in their methods, and no previous study has sought to assess whether their thermal outcomes are also similar.

**Methods:**

We controlled environmental variables and measured the maximum temperatures and temperature changes of a silicon phantom in which K-type thermocouples were embedded at depths of 0, 2, 5, 7, and 10 mm. WA and HA were also performed with acupuncture needles of various thicknesses (0.30 × 40 mm, 0.40 × 40 mm, and 0.50 × 40 mm).

**Results:**

Different time-dependent temperature distributions were observed between the two acupuncture methods: HA yielded a higher maximum temperature and temperature change on the surface, whereas WA yielded higher temperatures at the other tested depths. The thermal patterns were similar among the needles of different thicknesses for each method, with the following exception: while the temperature change and maximum temperature did not differ significantly by needle thickness for WA, these parameters increased significantly with needle thickness for HA.

**Conclusion:**

The two acupuncture procedures yielded different thermal patterns in a controlled environment. Further studies are necessary to reflect the effect of external environment variables occurring in reality.

## 1. Introduction

Warm-needle acupuncture (WA) is a therapy in which acupuncture and moxibustion are combined by stimulating acupoints with needles whose handles are affixed with burning pieces of moxa. [1, 2] WA is often used to treat painful conditions, such as osteoarthritis, rheumatoid arthritis, lumbar disc herniation, and musculoskeletal pain diseases. [3–6] The therapeutic mechanism of WA is similar to the process of thermotherapy, in which a high-temperature area is formed locally in a tissue to promote metabolism, enlarge blood vessels, and reduce the excitability of peripheral nerves. [7] WA has been shown to stimulate deep tissue and warm the acupuncture points via both direct conduction through the needle shaft and convection/radiation from the burning moxa. [8, 9]

In contrast to WA, fire-needle acupuncture (FA) involves the rapid insertion and withdrawal of red-hot needles. [10] The FA exhibits high-temperature resistance, and the needle retention time of FA is less than that of WA. [11] A new version of FA, termed “heated-needle acupuncture” or “heating-after-insertion FA” (HA), was recently described and conducted in Korea, wherein the needle is inserted and then directly heated. [12] HA is reported to be effective for treating damage to dense connective tissues, including ligaments, fibrous articular capsules, and tendons. [13]

Both WA and FA involve the application of heat stimulation to the needle, but there is a difference in the procedure: In WA, moxa is burned after insertion, whereas in FA, the needle is heated with fire before insertion. [12] The distinction of heating method between WA and HA is less clear, as both methods involve the application of heat after needle insertion. The differences between the two acupuncture methods lie in the heat source and heating time. In WA, the needle is heated for a long time with burning moxa, and in HA, the needle is directly heated for a shorter time by fire. Choi et al. suggested that WA and HA should be considered different treatments because the needle temperature varied depending on whether moxa combustion or match fire was used, noting that the firepower strength was higher for moxa than match fire. [14] However, Choi et al. studied only the temperature of a noninserted needle and did not seek to control the measurement environment.

Although several studies have examined temperature outcomes during WA [15], no such work has been reported for HA. In addition, most of the studies on WA have measured temperature only at or above the surface. There is a general lack of research on how the heat energy transferred through needles is distributed within tissues.

In this study, we compared the thermal distributions during WA and HA by measuring the temperature at several depths in an insertion medium while controlling the external environment and further evaluated the effect of needle thickness on the results obtained from each acupuncture method.

## 2. Materials and Methods

### 2.1. Materials

**Figure 1:**
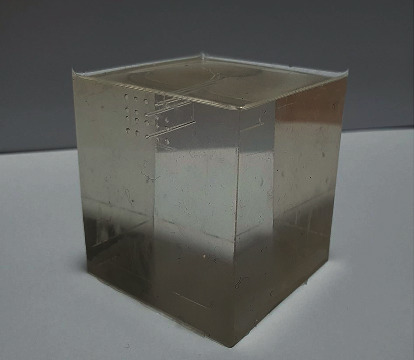
Fabricated phantom.

**Figure 2:**
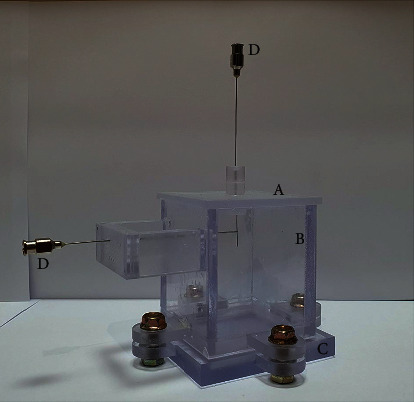
Polycarbonate mold and spinal needles: (A) lid, (B) main body, (C) base plate, and (D) needles.

**Figure 3:**
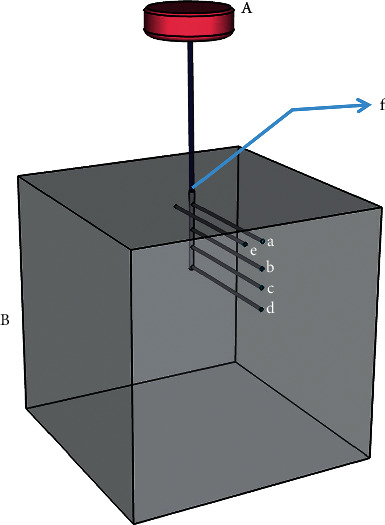
Schematic representation of the test specimen: (A) moxa (when used) and (B) phantom (thermocouple junction located at (a) 2 mm deep, (b) 5 mm deep, (c) 7 mm deep, (d) 10 mm deep, (e) 2 mm deep and 3 mm from the centerline axis, and (f) 0 mm (exposed surface)).

A silicon phantom consisting of polydimethylsiloxane (Sylgard 184; Dow Corning, USA) was prepared (Figure 1). The base and curing agent were combined at a 10 : 1 volumetric ratio, sufficiently mixed at room temperature (25∼26°C), and placed in a polycarbonate mold (frame: 5 cm wide, 5 cm long, and 6.3 cm high; phantom: 5 cm wide, 5 cm long, and 5 cm high). The mold consisted of a main body, lid, and base plate, three of which are assembled and screwed while the phantom is hardening and separated for easy extraction. The main body had several holes depending on the depth, so spinal needles (20G × 89 mm, Hakko, Japan) were located at the depth to be measured, creating spaces for the thermocouple in the hardened phantom. Outside the main body, there was a supporting part with a hole at the same height so that the spinel needles could be horizontally fixed during the phantom formation process. In the lid of the mold, there was a hole located in the center, through which a spinal needle (26G × 89 mm, Hakko, Japan) for the acupuncture needle space was located (Figure 2). Each hole was positioned as follows: the needle hole was located at the vertical center of the phantom and 1 cm deep. The thermocouple holes were made horizontally, oriented to contact the center-inserted needle at depths of 2 mm, 5 mm, 7 mm, and 10 mm from the exposed phantom surface; an additional thermocouple hole was also made 3 mm horizontally from the center needle at a depth of 2 mm (Figure 3). Each phantom was degassed using a vacuum pump (MVP-12; Woosung Automa Co., Korea) and chamber assembly, and then cured for 45 minutes in a forced-convection oven (OF-02 GW; Jeiotech Co., Korea) at 100°C. After the phantom hardened, the spinal needles were removed, the thermocouple and acupuncture needle were inserted, and then temperature measurements were performed.

The fabricated phantom consisted of dimethyl, xylene, ethylbenzene, dimethylvinyl-terminated dimethyl siloxane, di-methylvinylated and trimethylated silica, trimethylsiloxy silane, methylhydrogen siloxane, and tetramethyl tetravinyl cyclotetrasiloxane, which are substances contained in the base and curing agent [16].

When attempting to form all five thermocouple holes simultaneously, the thickness of the needle generated a placement error. Thus, experiments were conducted using two phantom groups: one with thermocouple holes at 2 mm, and 7 mm on the center line; and one with thermocouple holes at 5 mm, 10 mm on the center line and 2 mm offset by 3 mm from the center line.

Disposable stainless steel acupuncture needles (Dongbang Co., Korea) were used. Each acupuncture method was compared with needles of three different diameters: 0.30 × 40 mm, 0.40 × 40 mm, and 0.50 × 40 mm. For WA, a smokeless moxa of 12 mm in diameter, 5 mm in height, and 0.3 g in weight (Dongbang Ae moxa device; Dongbang Co., Korea) was used as the heating source.

### 2.2. Methods

#### 2.2.1. Controlling the Environment

**Figure 4:**
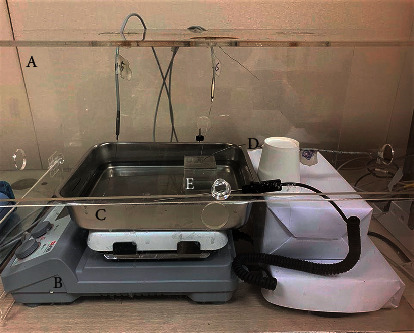
Setup of the temperature measurement environment: (A) acryl box, (B) hot plate, (C) tray containing water, (D) thermocouple, and (E) phantom with needle and moxa.

The experimental setting was designed to minimize the impact of the external environment and make the temperature distribution of the phantom similar to that of skin and deep tissue. The set-up consisted of an acrylic box (60 cm wide, 34 cm long, and 35 cm high), a hot plate, and a tray containing water (Figure 4).

Before acupuncture procedures, the phantom was placed in a convection oven for 30 minutes at 40°C to heat the surface temperature to 30∼31°C. A tray containing hot water (38∼42°C) was placed on the hot plate, and the lower part of the preheated phantom was submerged so the surface temperature of the phantom was maintained at 30 ∼ 31°C. The sides and top of the tray were enclosed within the acrylic box, and a heating fan was used to maintain the air temperature inside the box at 27∼29°C. When each presetting was maintained at the preset temperature for 20 seconds on the real-time temperature change graph, simulated acupuncture tests were conducted.

By presetting this apparatus at the temperature of human skin (30∼31°C) [17], we could indirectly predict the temperature changes that would be induced at different depths of human tissue by the two distinct acupuncture procedures.

#### 2.2.2. Performing Acupuncture

A needle was inserted into the phantom at a depth of 1 cm, and thermocouples were positioned at 0 mm (exposed surface), 2 mm, 5 mm, 7 mm, and 10 mm below the surface, in line with the needle; and at 2 mm below the surface, offset 3 mm horizontally from the center.

For WA, a moxa was located 33 mm above the surface. When it was maintained under the aforementioned conditions, the upper side of moxa was heated, and a needle was ignited using a lighter (YHL-8017; CIXI S.K., China). The temperature was measured at each thermocouple until the combustion was complete. Each experiment was repeated 10 times.

HA was conducted as described above, except that the needles were heated directly by applying a lighter to the needle at 10 mm above the surface. According to a review of FA [12], previous studies applied heat until the patient reported feeling a hot or stinging sensation, or for 1∼4 seconds past that point. We determined the heating time based on the results of preliminary experiments showing that an intolerable heat sensation occurred at an average of 5 seconds of heating for 0.30 × 40 mm needles and 4 seconds of heating for thicker needles. In preliminary experiments, five adult men and women were given HA on their forearms five times each with different thicknesses of needle and checked the bearable time, averaging each of the 25 data to obtain the heating time.

To compare the temperature changes as a function of needle thickness, acupuncture was performed as described above, with the needle heated for 4 seconds. The temperature was measured at each thermocouple during 130 seconds, and each experiment was repeated 10 times. We excluded the highest and lowest temperatures and averaged the remaining eight data points to protect the median against outliers.

#### 2.2.3. Measuring Temperature Changes

Temperature was measured using K-type thermocouples (Ø 0.5 mm; Shinsegi Sensor, Korea), which have a general temperature range of −200°C to 1260°C and special limits of error of ±1.1°C or 0.4% above 0°C. [18] In thermocouple, when two different metals (chromel and aluminum in the case of K-type) at two junctions made a closed circuit, and the junctions are maintained at different temperatures, an electromotive force is induced in this closed circuit. The amount of induced electromotive force is proportional to the temperature difference of the junctions, so the temperature is measured by electromotive force through a DC voltmeter between two junctions. [18].

Seven thermocouples were positioned as follows: at the exposed surface; at depths of 2 mm, 5 mm, 7 mm, and 10 mm from the surface, contacting the inserted needle; and at 3 mm from the inserted needle at a depth of 2 mm. The air temperature inside the acrylic box and the water temperature in the tray were also monitored.

Analog signals transmitted from the seven thermocouples were sent to the data processing program via the data acquisition device, which consisted of two NI 9211 modules (National Instruments, USA) inserted into an NI cDAQ-9174 Compact DAQ chassis (National Instruments, USA). The electric signal was converted into a digital signal through NI cDAQ-9174 Compact DAQ chassis, and data were plotted a graph on the computer screen and stored the numerical data through LabVIEW (National Instruments, USA). Temperatures were recorded at intervals of 1 second. Temperature data at each measuring point were processed using Excel (Microsoft, USA).

### 2.3. Statistical Analysis

All data are presented as mean ± standard deviation. Data analysis and graphical representations were generated using GraphPad Prism 7 software (GraphPad Software Inc., USA). The temperature change at each depth was compared with the mean value of the change (maximum temperature–base temperature). Statistical comparisons between values from WA and HA were performed using either the Student's *t*-test or the Mann–Whitney *U* test, as deemed appropriate according to the data distribution. Statistical comparisons between values from WA or HA by needle thickness were made using one-way ANOVA test or Kruskal–Wallis test, depending on the data distribution. If the results indicated statistical significance, a posttest was conducted with Tukey's HSD. *P* values < 0.05 were considered significant.

## 3. Results

### 3.1. Thermal Change Patterns Relative to Sensor Depth

#### 3.1.1. WA

**Figure 5:**
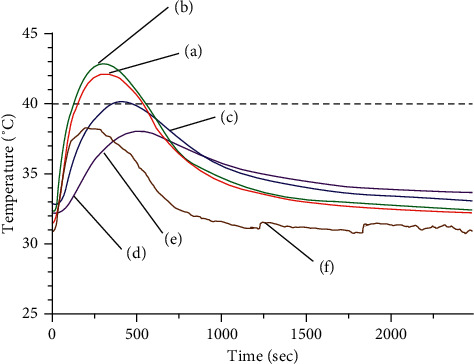
Thermal distribution at (a) 2 mm deep, (b) 2 mm deep and 3 mm horizontally from center, (c) 5 mm deep, (d) 7 mm deep, (e) 10 mm deep, and (f) phantom surface, during warm-needle acupuncture (WA).

The temperature change observed following moxibustion was larger inside the phantom than outside. Inside the phantom, the temperature and its rate of change both decreased with increasing depth (see Figure 5). The highest maximum temperatures were recorded at a position 2 mm deep (42.35 ± 0.715°C) and at 2 mm deep and 3 mm away from center (42.92 ± 1.128°C). The recorded temperature gradually decreased to 40.24 ± 0.497°C at 5 mm depth, 38.12 ± 0.567°C at 7 mm depth, and 37.35 ± 0.386°C at 10 mm depth. The mean change in temperature was also the largest at a depth of 2 mm (10.91 ± 0.680°C at the center, 10.53 ± 1.251°C at 3 mm from the center). As the depth in the phantom increased, an increasingly smaller change was registered: 7.33 ± 0.575°C at 5 mm depth, 5.92 ± 0.685°C at 7 mm depth, and 3.76 ± 0.412°C at 10 mm depth.

At the exposed surface, the maximum temperature was 37.64 ± 0.824°C, and the mean temperature change was 7.66 ± 0.768°C.

#### 3.1.2. HA

**Figure 6:**
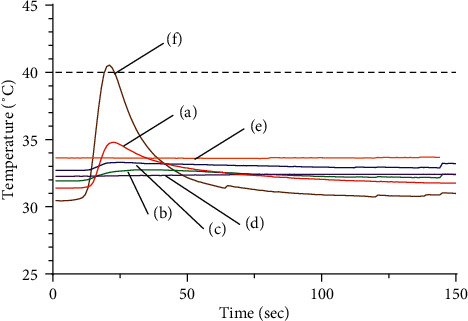
Temperature distribution at (a) 2 mm deep, (b) 2 mm deep and 3 mm horizontally from center, (c) 5 mm deep, (d) 7 mm deep, (e) 10 mm deep, and (f) phantom surface, during heated-needle acupuncture (HA).

The mean temperature change was the largest at the surface, while the deeper measurement points showed smaller temperature changes (see Figure 6). The temperature changed less than 1°C per position below the 5 mm depth. The maximum temperature and the mean change of temperature were 40.85 ± 0.969°C and 10.42 ± 1.074°C, respectively, at the surface and 37.94 ± 1.019°C and 3.54 ± 1.038°C, respectively, at the thermocouple located 2 mm deep. The thermocouple located 2 mm deep and 3 mm from the center showed a maximum temperature of 32.86 ± 0.281°C and a mean difference of only 0.93 ± 0.374°C.

#### 3.1.3. Comparing Thermal Distribution between WA and HA

The two procedures exhibited quite different patterns of temperature change: WA generated an S-curved graph; it showed an initial slow rise after ignition that shifted to a rapid rise, reached a peak, dropped rapidly, and then decreased more slowly. The HA graph, in contrast, exhibited a rapid rise during the heating period followed by a sharp decline occurring once the heat source was withdrawn.

**Figure 7:**
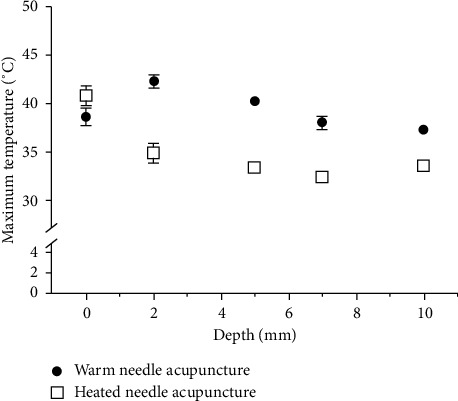
Comparison of the maximum temperatures achieved by warm-needle acupuncture (WA) and heated-needle acupuncture (HA).

**Figure 8:**
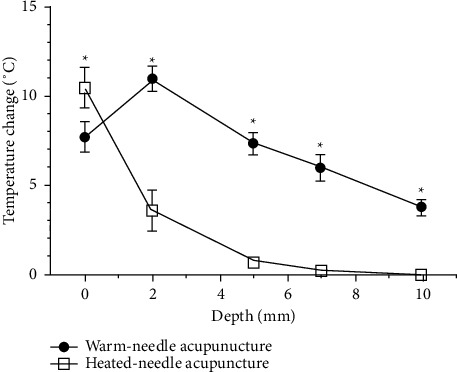
Comparison of the temperature changes generated by warm-needle acupuncture (WA) and heated-needle acupuncture (HA). Temperature changes were obtained as the mean of differences between the maximum and baseline temperatures. ^*∗*^A significant difference (*P* < 0.01).

Regarding the maximum temperature and the mean temperature change at each depth (see Figures 7 and 8), the temperature at all depths except at the surface was higher in WA than HA. Between the two acupuncture methods, the temperature change at the surface was significantly greater for HA than WA (*P* < 0.01), while those at 2 mm, 5 mm, 7 mm, and 10 mm depths were significantly greater for WA than HA (*P* < 0.01). In particular, the temperatures registered during HA changed less than 1°C between each measurement point deeper than 5 mm, whereas those registered during WA were 7.33 ± 0.575°C at 5 mm depth, 5.92 ± 0.685°C at 7 mm depth, and 3.76 ± 0.412°C at 10 mm depth.

Based on previous studies [19, 20], we considered 40∼45°C to represent the therapeutic temperature window and defined the therapeutic time as the period during which the temperature obtained during WA or HA was within this window. WA showed temperatures within this window at the 2 mm depth, 2 mm deep, and 3 mm horizontally from center and 5 mm depth points with therapeutic times of 404.3 ± 65.55 s, 406.75 ± 112.46 s, and 152.4 ± 128.5 s, respectively. In contrast, HA registered a temperature exceeding 40°C only at the phantom surface, with a duration of only 3.75 ± 2.99 s.

### 3.2. Comparing Thermal Outcomes by Needle Thickness

#### 3.2.1. WA

**Figure 9:**
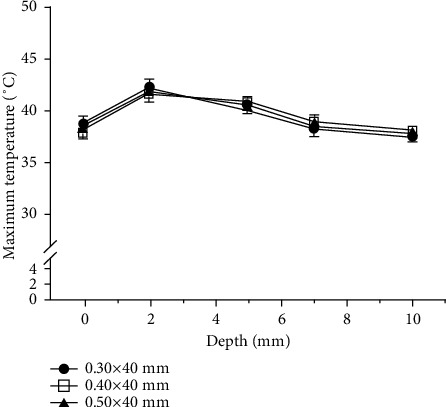
Comparison of the mean maximum temperature by needle thickness for warm-needle acupuncture (WA).

**Figure 10:**
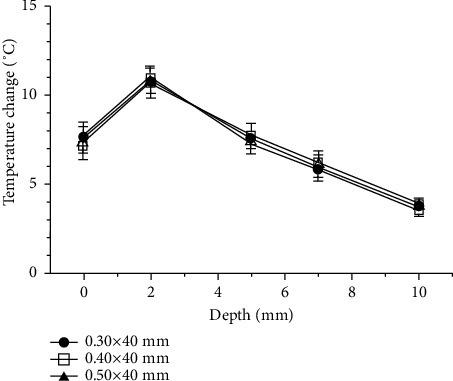
Comparison of the mean temperature change by needle thickness for warm-needle acupuncture (WA). Temperature changes were obtained as the mean of the differences between the maximum and baseline temperatures.

WA yielded the same pattern of depth-related temperature change regardless of needle thickness. All three needles tested yielded the highest maximum temperature and largest temperature change at a depth of 2 mm, with the maximum temperature and temperature change gradually decreasing with increasing depth. The maximum temperatures obtained using needles of three different thicknesses at each depth differed by less than 1°C (Figure 9), and the mean temperature change was not significantly different (Figure 10).

The needles of all three thicknesses yielded therapeutic temperatures at depths of 2 mm and 5 mm for WA. The therapeutic durations were 404.3 ± 65.55 s (0.30 × 40 mm), 340.4 ± 74.24 s (0.40 × 40 mm), and 410 ± 77.82 s (0.50 × 40 mm) at 2 mm depth and 152.4 ± 128.5 s (0.30 × 40 mm), 232.6 ± 196.8 s (0.40 × 40 mm), and 144.8 ± 148.7 s (0.50 × 40 mm) at 5 mm depth. There was no significant thickness-related difference observed in this parameter.

#### 3.2.2. HA

**Figure 11:**
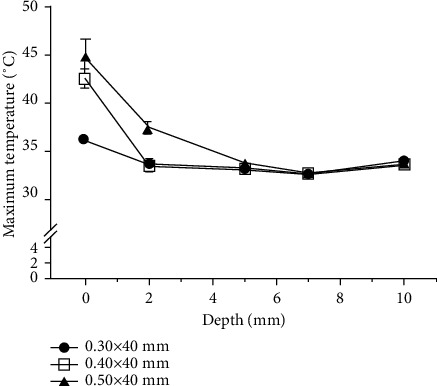
Comparison of the mean maximum temperature by needle thickness for heated-needle acupuncture (HA).

HA yielded the same pattern of depth-related temperature change regardless of needle thickness, with the largest variation seen at the surface and the temperature change decreasing in magnitude as the depth increased. At the surface, the maximum temperature increased with needle thickness, registering at 44.91 ± 1.77°C for the 0.50 × 40 mm needle, 42.61 ± 0.90°C for the 0.40 × 40 mm needle, and 36.14 ± 0.43°C for the 0.30 × 40 mm needle. At 2 mm depth, the maximum temperature was the highest for the 0.50 × 40 mm needle (37.49 ± 0.66°C) followed by the 0.30 × 40 mm and 0.40 × 40 mm needles at 33.69 ± 0.23°C and 33.41 ± 0.70°C, respectively. At depths below 5 mm, the maximum temperatures did not differ by more than 1°C across the tested needle thicknesses (Figure 11).

**Figure 12:**
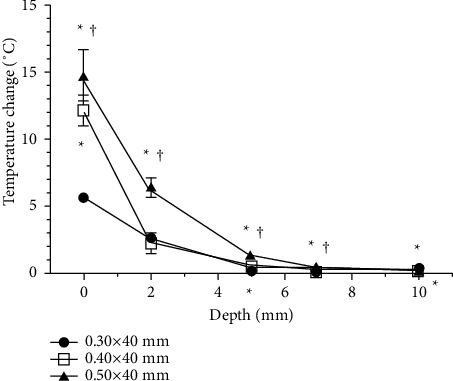
Comparison of the mean temperature change by needle thickness for heated-needle acupuncture (HA). Temperature changes were obtained as the mean of the difference between the maximum and baseline temperatures. ^*∗*^A significant difference (*P* < 0.01) compared to the 0.30 × 40 mm needle; ^†^a significant difference (*P* < 0.01) compared to the 0.40 × 40 mm needle.

The measured temperature change was largest with the 0.50 × 40 mm needle at all depths; this difference was significant for the 0.30 × 40 mm needle at all depths, and for the 0.40 × 40 mm needle at all depths except for 10 mm. When comparing the 0.30 × 40 mm needle with the 0.40 × 40 mm needle, the temperature changes obtained using the 0.40 × 40 mm needle were significantly larger at the surface and at depths of 5 mm and 10 mm. Generally, however, the temperature changed less than 1°C at depths below 5 mm for the 0.30 × 40 mm and 0.40 × 40 mm needles and at depths below 7 mm for the 0.50 × 40 mm needle (Figure 12).

The surface temperatures obtained using the 0.40 × 40 mm and 0.50 × 40 mm needles were within the therapeutic temperature window, with therapeutic times of 8.86 ± 1.45 s and 14.38 ± 2.55 s, respectively.

## 4. Discussion

WA and HA are combined treatments that apply heat via acupuncture treatment, with the goal of transferring heat deep into the human body. Conventional FA is performed by quickly inserting and withdrawing a red-hot needle at an acupuncture point; HA resembles this by using a relatively strong heat stimulation. However, HA resembles WA in that the application of heat stimulation after needle insertion. It could therefore be inappropriate to simply classify HA as a version of WA or FA. Moreover, the literature lacked any study seeking to differentiate the thermal distribution during HA or WA.

The existing research on temperature outcomes obtained by WA has mainly focused on temperature changes at or above the skin surface [21–23]. Moreover, even though the thermal efficiency of WA is known to be greatly affected by environmental factors, such as airflow [24], the external environment was often not controlled in previous studies. [15] Accordingly, we herein sought to investigate the thermal outcomes of WA and HA at various depths of a model system with a controlled external environment. We also assessed the effect of acupuncture needle thickness.

Heat applied to the skin stimulates cutaneous thermoreceptors, resulting in therapeutic effects via the activity of neuronal fibers. [25] Thermal sensation is mediated by various primary afferent nerve fibers that transduce, encode, and transmit thermal information. Most of these afferents have characteristic reaction functions that enable them to encode the intensity of the stimulus. Warm fibers, which consist of the so-called “C fibers,” are active at temperatures above 30°C, exhibit their maximum discharge at 40∼43°C, and have sharply reduced responses at higher temperatures. However, a heat stimulus beyond this threshold (43∼47°C) is perceived by nociceptor “A fibers” as a distinct pain sensation. When the stimulus temperature increases, therefore, the sensation may turn from warmth to heat to pain. [26, 27].

In order for athermal stimulus to produce therapeutic effects, it must reach a certain temperature window. A temperature over 40°C is required to produce physiological effects, such as elevating metabolism, dilating blood vessels, and increasing peripheral blood flow, but heat-pain sensation occurs at 44.4 ± 2.1°C (with some individual differences) and tissue damage occurs when the temperature is maintained at over 45°C. [28–30] Therefore, in this study, we set the therapeutic temperature window at 40°C to 45°C.

Our results showed that WA heated the needle to within the therapeutic temperature window at depths of 2 mm and 5 mm, while HA achieved this effect only at the exposed surface. In human tissue, the epidermis (the outermost layer of the skin) is approximately 40 ∼ 70㎛ thick; below that lies the dermis, which is 0.5∼4 mm thick and thus 15∼40 times thicker than the epidermis. The epidermis and dermis contain numerous cutaneous receptors, including mechanoreceptors, thermoreceptors, and nociceptors. [31] Previous studies estimated that the heat-sensitive receptors of C fibers are located in the epidermis and dermis at depths ranging from 20 to 600 *μ*m. [32, 33] However, Ivanov et al. suggested that thermoreceptors are not only distributed in the superficial layers of the skin but they are also located in deeper layers. [34] This suggests that therapeutic effects could be obtained from techniques that can reach the therapeutic temperature window not just at the surface (corresponding to the epidermis) but also at depths of 2 mm and 5 mm (corresponding to the dermis). We herein found that WA showed larger temperature changes at these depths, suggesting that WA provides more sufficient thermal stimulation.

Our analysis of the maximum temperature and temperature change with respect to needle thickness revealed that WA and HA yielded different results: while the temperature change and maximum temperature did not differ significantly as a function of needle thickness for WA, these parameters increased significantly with needle thickness for HA. Previous studies on the mechanism of WA suggested that heat is transferred to the tissue both by conduction through the needle and by convection and radiation from the burning moxa. However, the stainless steel needle that is mainly used in clinical practice has a low thermal conductivity, meaning that convection and radiation from the moxa play major roles in the thermal effect of WA. [35, 36] As various factors have been shown to change the magnitude of this thermal stimulation, such as the ignition position of the moxa [37], the distance between the moxa and the skin surface [38], and the wind velocity of the experimental environment [24], it is notable that we controlled various external environmental factors and found that needle thickness did not significantly affect the thermal outcome of WA.

For HA, in contrast, the 0.40 × 40 mm and 0.50 × 40 mm needles were found to yield thermal outcomes within the therapeutic temperature window. Notably, the mean surface temperature reached by the thickest needle (0.50 × 40 mm) was 44.91 ± 1.77°C, which is close to the temperature that can cause pain sensation or tissue damage. Thus, practitioners should take care when applying needles of this size in clinical practice.

The core temperature of burning moxa, which generally depends on its density and weight, is 600∼700°C [39], whereas the core temperature of the lighter flame used as the heating source for HA is 1000∼1200°C. [40] This difference in core temperature suggests that HA could exhibit more heat conduction through the needle, compared to WA, which could account for the needle-thickness-dependent difference in the predicted therapeutic effect.

This study has several limitations. First, as a human skin replacement, we herein used Sylgard 184, which is commonly used to make vascular models in mechanobiology studies [41, 42]. The human body comprises fat, protein, body fluids, and blood flow and has its own thermoregulatory mechanisms to induce cooling of regions exposed to excessive external heat. These aspects were not reproduced in the phantom. In several studies [43, 44], a tissue model of Yucatan minipig that is anatomically similar to human skin is used as a human skin replacement for skin absorption and wound healing experiments. However, it is difficult to locate thermocouples at exact depths, and also does not reflect the thermoregulatory mechanisms of the human body. In this study, the phantom is designed to resemble the temperature distribution of human skin and deep tissues, although less similar to human tissues. Previous studies [45, 46] have reported that the body temperature rises by about 1°C as it enters the epidermis, dermis, and subcutaneous tissues. Therefore, in this study, the base temperature was set higher by 1°C at 7 mm and 10 mm (corresponding to subcutaneous fat) than at 2 mm and 5 mm (corresponding to the dermis), by submerging the lower part of this phantom in 38 ∼ 42°C water. Second, we focused on observing the temperature variations obtained within the tissue model for both acupuncture methods while controlling environmental factors. [38] Further studies are warranted to assess the effect of external environmental factors occurring in the clinic. Third, we did not assess the temperature outcomes of traditional FA. This is because FA is performed temporarily, without retention of the needle within the tissue, making it difficult to measure the temperature or control the external environment. A previously reported temperature measurement of FA [47] monitored only a noninserted needle, and thus the prior findings could not be compared with our present results on WA and HA.

Despite these limitations, the present work is a meaningful first step in comparing the thermal distribution of WA and HA. They at first appear to be very similar in their methodologies, but we herein show that they yield very different temperature outcomes, with WA offering a longer therapeutic time than HA. We also show that the applied thermal stimulation may vary depending on the needle thickness in HA, indicating that practitioners should be careful to choose a needle of a thickness that is both effective and safe. Going forward, additional studies are needed to assess these temperature outcomes using skin replacement models that more closely reflect the properties of real human skin.

## Data Availability

The data and material from this trial are available upon reasonable request and approval by the corresponding author.

## References

[1] Qi L. Z., Ma X. P., Honh J. (2013). Comparison of moxibustion materials and methods between ancient and modern times. *Chinese Arch Traditional Chinese Medicine*.

[2] Yu J. (2015). The rise of Chinese acupuncture in the west: how an ancient eastern tradition became an American medical staple. *Vet Hert*.

[3] Wang J. G., He L. J. (2007). Observation on the therapeutic effect of warming needle moxibustion on knee osteoarthritis. *Zhongguo Zhen Jiu*.

[4] Kim Y. J. (2017). Clinical observation on warm acupuncture therapy for rheumatoid arthritis. *Journal of Traditional Medicine and Clinical Naturopathy*.

[5] Li X., Han Y., Cui J., Yuan P., Di Z., Li L. (2016). Efficacy of warm needle moxibustion on lumbar disc herniation. *Journal of Evidence-Based Complementary & Alternative Medicine*.

[6] Chung J. Y., Choi D. Y., Woo H. S., Kang S. K. (2009). Review of clinical trials on warming acupuncture for musculoskeletal pain diseases: a systematic review. *J Acupunct Res*.

[7] Yang L., Tan J.-Y., Ma H. (2018). Warm-needle moxibustion for spasticity after stroke: a systematic review of randomized controlled trials. *International Journal of Nursing Studies*.

[8] Cheng K., Ding W., Shen X., Ding G. (2007). Study of heat conduction of warming acupuncture. *Shanghai Journal of Acupuncture Moxibustion*.

[9] Tzou C.-H. J., Yang T.-Y., Chung Y.-C. (2015). Evaluation of heat transfer in acupuncture needles: convection and conduction approaches. *Journal of Acupuncture and Meridian Studies*.

[10] Wang H.-r. (2017). Characteristics and key to manipulation of fire needle. *World Journal of Acupuncture-Moxibustion*.

[11] Li S., Xie P., Liang Z. (2018). Efficacy comparison of five different acupuncture methods on pain, stiffness, and function in osteoarthritis of the knee: a network meta-analysis. *Evidence-based Complementary and Alternative Medicine*.

[12] Moon S. J., Kong J. C., Jo D. C., Kim E., Song Y. S., Lee J. H. (2011). Review of studies on fire needle. *J Oriental Rehab Med*.

[13] Oh S. G. (2011). *Stimulation Therapy*.

[14] Choi G. M., Eom T. S. (1992). Effect of the quality of acupuncture on variation of temperature in the warming needle. *J Acupunct Res*.

[15] Lee J. H., Jo H. R., Kim S. H. (2019). A review on the characteristics of temperature variation in warm needle. *Journal of Korean Medicine*.

[16] Palchesko R. N., Zhang L., Sun Y., Feinberg A. W. (2012). Development of polydimethylsiloxane substrates with tunable elastic modulus to study cell mechanobiology in muscle and nerve. *PLoS One*.

[17] Chun S. I., Park E. S., Yi C. H. (1995). Digital infrared thermal imaging on normal healthy subjects. *J Korean Acad Rehabil Med*.

[18] Committee E. (1993). *Manual on the Use of Thermocouples in Temperature Measurement (4th)*.

[19] Gao X. Y., Chong C. Y., Zhang S. P., Cheng K. W. E., Zhu B. (2012). Temperature and safety profiles of needle-warming techniques in acupuncture and moxibustion. *Evidence-based Complementary and Alternative Medicine*.

[20] Yi S.-H. (2009). Thermal properties of direct and indirect moxibustion. *Journal of Acupuncture and Meridian Studies*.

[21] Kim J. W., Lee H. J., Ahn C. B., Yi S. H. (2011). Study on the thermal properties of warm needle and the development of warm needle apparatus. *J Acupunct Res*.

[22] Huang V. C., Sheu T. W. (2008). Heat transfer involved in a warm (moxa-heated) needle treatment. *Acupuncture & electro-therapeutics research*.

[23] Zhao Y., Qin Y., Zheng J. (2012). Discussion on the temperature characteristic of silver needle in the human body during the warm needling. *Chinese Acupuncture & Moxibustion*.

[24] Kim J. W., Lee H. J., Yi S. H. (2010). Study of air flow effects on heat characteristics of warm needle acupuncture. *Korean Journal of Acupuncture*.

[25] Wall P. D. (2002). *Text Book of Pain*.

[26] Schepers R. J., Ringkamp M. (2010). Thermoreceptors and thermosensitive afferents. *Neuroscience & Biobehavioral Reviews*.

[27] Dubin A. E., Patapoutian A. (2010). Nociceptors: the sensors of the pain pathway. *Journal of Clinical Investigation*.

[28] Menétrey D., Chaouch A., Besson J. M. (1979). Responses of spinal cord dorsal horn neurons to non-noxious and noxious cutaneous temperature changes in the spinal rat. *Pain*.

[29] Defrin R., Ohry A., Blumen N., Urca G. (2002). Sensory determinants of thermal pain. *Brain: A Journal of Neurology*.

[30] LaMotte R. H., Campbell J. N. (1978). Comparison of responses of warm and nociceptive C-fiber afferents in monkey with human judgments of thermal pain. *Journal of Neurophysiology*.

[31] Noh S. S. (2006). *Dermatology*.

[32] Tillman D. B., Treede R. D., Meyer R. A., Campbell J. N. (1995). Response of C fibre nociceptors in the anaesthetized monkey to heat stimuli: estimates of receptor depth and threshold. *Journal of Physiology*.

[33] Adair R. K. (1999). A model of the detection of warmth and cold by cutaneous sensors through effects on voltage-gated membrane channels. *Proceedings of the National Academy of Sciences*.

[34] Ivanov K., Konstantinov V., Danilova N., Sleptchuck N., Rumiantsev G. (1986). Thermoreceptor distribution in different skin layers and its significance for thermoregulation. *Journal of Thermal Biology*.

[35] Yeo S. (2013). The study on temperature measurement of warm needling using stainless steel needle and gold needle. *Korean Journal of Acupuncture*.

[36] Yang S.-B., Park S.-J., Lee J.-G., Jung J.-C., Kim J.-H. (2017). Experimental interpretation of heat transmits pattern on warm needling. *Korean Journal of Acupuncture*.

[37] Kim Y. H., Lee S. H., Yeo S. J., Choi I. H., Kim Y. K., Lim S. B. N. (2008). Study on ignition position-related changes in warm needle temperature. *Korean J Acupunct*.

[38] Yang S.-B., Kwon O. S. (2019). Principal components of thermal stimulation while the warm needling: diameter of the acupuncture needle and distance from the skin. *Korean Journal of Acupuncture*.

[39] Kim Y. H., Lee S. H., Yeo S. J., Choi I. H., Kim Y. K., Lim S. (2008). Study on moxa density-related changes in warm needle temperature. *Journal of Korean Oriental Medicine*.

[40] Vanhala P., Kurstjens D. A. G., Ascard J. Guidelines for physical weed control research: flame weeding, weed harrowing and intra-row cultivation.

[41] Bernardi L., Hopf R., Sibilio D., Ferrari A., Ehret A. E., Mazza E. (2017). On the cyclic deformation behavior, fracture properties and cytotoxicity of silicone-based elastomers for biomedical applications. *Polymer Testing*.

[42] Yousif M. Y., Holdsworth D. W., Poepping T. L. (2009). Deriving a blood-mimicking fluid for particle image velocimetry in Sylgard-184 vascular models. *Annual International Conference of the IEEE Engineering in Medicine and Biology Society. IEEE Engineering in Medicine and Biology Society. Annual International Conference*.

[43] Byl N. N., McKenzie A. L., West J. M., Whitney J. D., Hunt T. K., Scheuenstuhl H. A. (1992). Low-dose ultrasound effects on wound healing: a controlled study with Yucatan pigs. *Archives of Physical Medicine & Rehabilitation*.

[44] Nunoya T., Shibuya K., Saitoh T. (2007). Use of miniature pig for biomedical research, with reference to toxicologic studies. *Journal of Toxicologic Pathology*.

[45] Webb P. (1992). Temperatures of skin, subcutaneous tissue, muscle and core in resting men in cold, comfortable and hot conditions. *European Journal of Applied Physiology and Occupational Physiology*.

[46] Shrestha D. C., Acharya S., Gurung D. B. (2020). Modeling on metabolic rate and thermoregulation in three layered human skin during carpentering, swimming and marathon. *Applied Mathematics*.

[47] Shao J. X. (2015). *Study on the Temperature Measurement and Numerical Simulation of the Fire Needle*.

